# Development of cellular models expressing cynomolgus (*Macaca fascicularis*) HER2 for the functional evaluation of cross-reactive anti-human HER2 response

**DOI:** 10.3389/fphar.2025.1675875

**Published:** 2025-11-13

**Authors:** Chiara Cappello, Francesca Ruzzi, Julia Consoli, Maria Sofia Semprini, Laura Scalambra, Olga Maria Pittino, Stefania Angelicola, Arianna Palladini, Mette Thorn, Patrizia Nanni, Pier-Luigi Lollini

**Affiliations:** 1 Department of Medical and Surgical Sciences (DIMEC), University of Bologna, Bologna, Italy; 2 Center of Applied Biomedical Research (CRBA), University of Bologna, Bologna, Italy; 3 IRCCS Azienda Ospedaliero-Universitaria di Bologna, Bologna, Italy; 4 Department of Molecular Medicine, University of Pavia, Pavia, Italy; 5 Medical Oncology Division, Fondazione IRCCS Policlinico San Matteo, Pavia, Italy; 6 Capital Region Pharmacy, Herlev, Denmark

**Keywords:** breast cancer, HER2, anti-HER2 therapies, *in vitro* preclinical models, cross-reactivity studies

## Abstract

**Introduction:**

Resistance to anti-HER2 therapies and incomplete response remain considerable challenges in managing HER2-positive tumors. Furthermore, some healthy organs, like the heart, express low levels of HER2, entailing a risk of toxic side effects, such as cardiotoxicity. Thus, the development of new anti-HER2 agents, to improve therapy outcomes, is still ongoing and requires preclinical evaluations of their side effects. Nonhuman primates are crucial in toxicology due to their high genetic similarity to humans. In line with the 3Rs principles, their use should be minimized by prioritizing the development of more predictive alternative methods. However, most in vitro assays (e.g., ELISA) only show the binding of anti-HER2 agents to cynomolgus HER2, without revealing the functional activities, such as growth inhibition.

**Methods:**

We obtained cell lines expressing cynomolgus (*Macaca fascicularis*) HER2 (cyHER2), to evaluate the functional inhibitory activity of anti-human HER2 therapeutic agents on endogenous cynomolgus HER2 in three-dimensional (3D) growth condition *in vitro*.

**Results:**

Our model, based on NIH 3T3 cells, became sensitive to the monoclonal antibody trastuzumab and to the selective HER2 tyrosine kinase inhibitor tucatinib.

**Discussion:**

The results suggest that this model could be a promising tool for preclinical functional cross-reactivity tests of anti-HER2 therapies before *in vivo* studies.

## Introduction

Breast cancer is the most commonly diagnosed cancer and the leading cause of cancer-related death in women worldwide (Sedeta et al., 2023). Human epidermal growth factor receptor 2 (HER2) is overexpressed by 15%–30% of all breast cancer cases and its overexpression is associated with poor prognosis (Müller et al., 2023). The development of HER2-targeted therapies, including monoclonal antibodies (e.g., trastuzumab and pertuzumab), antibody–drug conjugates (ADCs) and tyrosine kinase inhibitors (TKIs) has revolutionized patient response rates and survival (Swain et al., 2023). However, many patients eventually develop intrinsic or acquired resistance, leading to disease progression (Rimawi et al., 2015; Choong et al., 2020; Guidi et al., 2023). Therefore, the development of new anti-HER2 agents remains essential, with rigorous evaluation of potential side effects being a critical priority. HER2 is primarily overexpressed by tumor cells, but it is also expressed at low levels in normal tissues, particularly in cardiomyocytes (Press et al., 1990; Fuchs et al., 2003), entailing a risk of cardiotoxicity following anti-HER2 therapy (Slamon et al., 2001; Dempsey et al., 2021; Gonciar et al., 2021). Cardiotoxic effects most frequently experienced by patients treated with anti-HER2 therapies include asymptomatic drops in left ventricular ejection fraction (LVEF), and left ventricular systolic dysfunction (LVSD), or congestive heart failure (CHF) (Gonciar et al., 2021; Pondé et al., 2016; Florido et al., 2017). Before human testing, tissue cross-reactivity studies are required for novel antibody-based therapeutic approaches to predict possible side effects on healthy tissues (Qu et al., 2020). In the field of anti-HER2 drug development, tissue cross-reactivity analyses are crucial to evaluate the effects of anti-HER2 treatments on normal tissues. Animal models, particularly rodents and nonhuman primates (NHP), remain the gold standard for predicting drug toxicity, as they provide a whole organism system that reflects complex biological interactions. Nonhuman primates, in particular macaques, sharing approximately 93% genetic homology with humans, are considered the most valuable model for evaluating cross-reactive responses of anti-HER2 drugs (Dewi and Cline, 2021). Moreover, NHP physiologically express HER2 in normal tissues (e.g., heart) like humans (Qu et al., 2020). Although animal models remain essential to evaluate drug efficacy and safety (Dorato and Buckley, 2007; Chang and Grieder, 2024), alternative methods are being explored to reduce animal use and to align with the 3Rs principle (MJA, 1960). *In vitro* techniques, such as enzyme-linked immunosorbent assays (ELISA) and immunometric tests, are commonly used to evaluate anti-HER2 monoclonal antibody binding (Koivunen and Krogsrud, 2006; Antos et al., 2024; Alhazmi and Albratty, 2023). However, these assays do not provide insights into functional activity—such as whether an antibody significantly inhibits (or enhances) cell growth. Therefore, we set up a cellular model for the functional evaluation of anti-HER2 agents binding the cynomolgus HER2.

Two-dimensional (2D) tumor cell cultures can be used to test anti-HER2 drugs. However, 2D cultures may show limited sensitivity to drugs and monoclonal antibodies if compared to three-dimensional (3D) *in vitro* assays (Hudziak et al., 1989). By better mimicking the cellular environment of living tissues, 3D cell growth assays may represent valid tools to obtain predictive and informative functional data on cross-reactivity and potential toxic effects of targeted therapies (Pampaloni et al., 2007).

Here, we present cellular models expressing cynomolgus monkey HER2 (cyHER2) evaluated in a 3D growth setting, to reveal the cross-reactivity of anti-HER2 antibodies and their functional activity against cynomolgus HER2 *in vitro*, before *in vivo* NHP studies.

## Materials and methods

### Cell lines

NIH 3T3 (fibroblastic cell line isolated from a mouse NIH/Swiss embryo) were a kind gift from Dr. M. Vaccari (Arpae, Emilia-Romagna). NIH 3T3 cell line and its derivatives were routinely cultured in Dulbecco’s Modified Eagle Medium (DMEM, Thermo Fisher Scientific, Monza, Italy) supplemented with 10% of fetal bovine serum (FBS, Thermo Fisher Scientific, Monza, Italy), 100 U/mL penicillin and 10 μg/mL streptomycin (Thermo Fisher Scientific, Monza, Italy).

CYNOM-K1 is a cell line established from cynomolgus monkey embryo skin, it was purchased from Istituto Zooprofilattico Sperimentale della Lombardia e dell'Emilia Romagna “Bruno Ubertini”, IZSLER, Brescia, Italy. CYNOM-K1 cell line and its derivatives were cultured in Eagle’s Minimum Essential Medium (EMEM, ATCC Manassas, Virginia) supplemented with 10% FBS, 100 U/mL penicillin, 10 μg/mL streptomycin and 1% Non-Essential Amino Acids (NEAA, EuroClone S. p.A. Pero, MI, Italy).

Human HER2-positive breast cancer cell line BT-474 (kindly provided by Dr. S. Pupa, Istituto Nazionale dei Tumori, Milan, Italy) was routinely cultured in RPMI (Thermo Fisher Scientific, Monza, Italy) supplemented with 10% FBS, 100 U/mL penicillin and 10 μg/mL streptomycin.

All cell lines were cultured at 37 °C in a humidified 5% CO_2_ atmosphere and were split once or twice a week according to density, using 0.05% trypsin–EDTA (Thermo Fisher Scientific). Cynomolgus monkey serum was purchased from Charles River Laboratories (Calco, Italy).

### Sequence alignment and homology prediction

The reference nucleotide sequences of human and cynomolgus HER2 were obtained from https://www.ncbi.nlm.nih.gov (accessed on 22 July 2022) and converted in aminoacidic sequence using the BLASTN tool (RRID: SCR_001598, https://blast.ncbi.nlm.nih.gov/Blast.cgi, accessed on 25 November 2024) setting as reference genome *Homo sapiens* and *Macaca fascicularis*, respectively (Altschul et al., 1990).

To analyze the degree of similarity between human and cynomolgus protein sequences, the Basic Local Alignment Search Tool (NCBI BLAST RRID: SCR_004870, accessed on 25 November 2024) was used. The identity percentage was calculated and visualized using the Pairwise Alignment function of Jalview software (RRID: SCR_0064599 (Waterhouse et al., 2009).

### Lentiviral particles and transfections

CyHER2 expression was obtained by transfection of NIH 3T3 and CYNOM-K1 cells with custom lentiviral particles with CMV promoter, C-DYKDDDDK C-terminal Epitope tag encoding Cynomolgus (*Macaca fascicularis*) HER2 gene sequence, produced by GenScript Biotech (Piscataway, NJ, United States) at a multiplicity of infection (MOI) of 100. A control with a commercial empty vector (pLenti-C-Myc-DDK-P2A-Puro, OriGene Technologies, Rockville, MD, United States) was run in parallel as transfection control. Polybrene (hexadimethrine bromide, Sigma Aldrich, Saint Louis, MO, United States) at a final concentration of 8 μg/mL was used to enhance transfection efficiency. Puromycin (Thermo Fisher Scientific) was added to the culture medium for antibiotic selection of transfected cells at the concentration of 0.5 μg/mL for CYNOM-K1-derived cell lines and 1 μg/mL for NIH 3T3-derived cell lines.

### Indirect immunofluorescence and flow cytometry

To evaluate HER2 expression level, cell lines were analyzed by immunofluorescence and cytofluorimetric analysis. Cells were dissociated to yield single-cell suspensions. Cells were then incubated with the following antibodies: mouse anti-human HER2 (clone MGR2, 14 μg/mL, Enzo Life Sciences, Lansen, Swisse Cat# ALX-804-573-C100, RRID:AB_2051117); mouse anti-human HER2 4D5 (14 μg/mL Genentech, South San Francisco, CA, United States); human anti-human trastuzumab (14 μg/mL, Herceptin, Genentech Roche Cat# 45-2317, RRID: AB_3669039), followed by the addition of secondary antibodies anti-mouse and human IgG (respectively AF488 Thermo Fisher Scientific Cat# A-11017 RRID:AB_2534084 and FITC-conjugated Thermo Fisher Scientific Cat# PA1-84339, RRID: AB_931552, at 20 μg/mL). Flow cytometry was performed with a CyFlow Space (Sysmex Partec, Goerlitz, Germany), and analyzed using FCS Express software (*De Novo* Software, Glendale, CA, United States RRID:SCR_016431).

### 3D-soft agar growth assay

*In vitro* sensitivity of cell lines to trastuzumab and tucatinib was evaluated under 3D growth conditions independent of anchorage to the substrate (soft agar).

As described in detail previously (Ruzzi et al., 2024), cells were seeded in semisolid complete culture medium +0.33% agar (Sea-Plaque Agarose, Lonza, Switzerland) on a 0.5% agar underlayer in 24-well plates (Corning Life Sciences, United States). NIH 3T3 cyHER2 were then tested with different concentrations of trastuzumab (10, 20 and 30 μg/mL, kindly provided by Genentech), tucatinib (10, 20, 100 and 1,000 nM, Selleck Chemicals LLC Houston, TX United States) and with male cynomolgus monkey serum (Charles River Laboratories, Calco, Italy) diluted 1:100.

Cells were seeded as follow: BT-474 at 500 cells/well in RPMI +10% FBS; NIH 3T3 and its transfectants at 500, 1,000, 5,000 or 10,000 cells/well in DMEM +10% FBS. For drug testing, the doses of 5,000 cells/well for NIH 3T3 cyHER2 and 1,000 cells/well for NIH 3T3 were chosen. CYNOM-K1 and its transfectants were seeded in MammoCult (Stemcell Technologies, Vancouver, Canada) supplemented with 1% FBS or EMEM +10% FBS at 500, 1,000, 5,000 or 10,000 cells/well.

Colonies (diameter >90 μm) were counted in an inverted microscope equipped with an ocular micrometer in dark-field, 11–27 days after cell seeding.

### Western blot analysis

Protein extraction and Western blotting were performed as reported previously (De Giovanni et al., 2014). The effect of trastuzumab and tucatinib on cells was evaluated after exposing cells to the treatment for 6 and 24 h. Untreated or vehicle samples ran in parallel as controls. The following primary antibodies were employed: anti-HER2 (clone 3B5, 0.1 μg/mL, Calbiochem, Merck, Darmstadt, Germany Millipore Cat# OP15L-100UG, RRID: AB_10681957), anti-pNeu (Tyr 1,248) (0.2 μg/mL, Santa Cruz Biotechnology, Santa Cruz, CA Cat# sc-293110, RRID: AB_10845875), anti-ERK1/2 (0.084 μg/mL, Cell Signaling Technology, Danvers, MA, Cat# 4695), anti-pERK1/2 (Thr202/Tyr204) (0.096 μg/mL, Cell Signaling Technology, Cat# 9101), anti-Actin (0.8 μg/mL, Merck, Darmstadt, Germany, Sigma-Aldrich Cat# A2066, RRID:AB_476693). Membranes were either incubated with polyclonal horse-radish peroxidase conjugated anti-rabbit IgG antibody (0.33 μg/mL, Bio-Rad Laboratories, Inc., Hercules, CA, United States Cat# 170-6515, RRID: AB_11125142), or anti-mouse IgG antibody (Bio-Rad Laboratories Cat# 170-6516, RRID: AB_11125547). Proteins were detected by chemiluminescent reaction (Clarity ECL Western blotting Substrates, Bio-Rad Laboratories) visualized using a ChemiDoc system (Bio-Rad Chemidoc XRS Gel Imaging System, Bio-Rad Laboratories, RRID: SCR_019690) and quantified through densitometric analysis of bands by Image Lab software Version 6.1.0 build 7 Standard Edition (Bio-Rad Laboratories, RRID:SCR_014210).

### Statistical analysis

All statistical analyses were performed using GraphPad Prism (GraphPad Software, Boston, Massachusetts United States, RRID: SCR_002798).

## Results

### Induction of functional cynomolgus HER2 expression in NIH 3T3 cell line

We employed the Basic Local Alignment Search Tool (https://blast.ncbi.nlm.nih.gov/Blast.cgi) and JalView software (Altschul et al., 1990; Waterhouse et al., 2009) to quantify and visualize similarity between human and cynomolgus HER2 sequences. We observed a high degree of similarity with an identity percentage of 98.25% between the amino acid sequence of the human (HumanHER2) and the cynomolgus (CynoHER2) HER2 (Supplementary Figures S1, S2).

NIH 3T3 fibroblasts are frequently used in carcinogenesis research, because of their sensitivity to oncogenic transformation (Hayashi et al., 2021; Rubin, 2017). Indeed, when NIH 3T3 are transfected with oncogenes, they are prone to lose contact inhibition and form multilayered foci, gaining the ability to grow in anchorage-independent way, which are hallmarks of tumorigenic potential (Clark et al., 1995). Moreover, transformed NIH 3T3 can be used in compounds screening for anti-cancer applications (Hayashi et al., 2021). Induction of cynomolgus (*Macaca fascicularis)* HER2 (cyHER2) expression in NIH 3T3 cells was obtained by transfection with lentiviral particles encoding cynomolgus *ErbB2* gene sequence. In parallel to cynomolgus HER2 transfection, NIH 3T3 cells were also transfected with an empty vector to obtain a negative transfection control (referred to as NIH 3T3 Vector).

**FIGURE 1:**
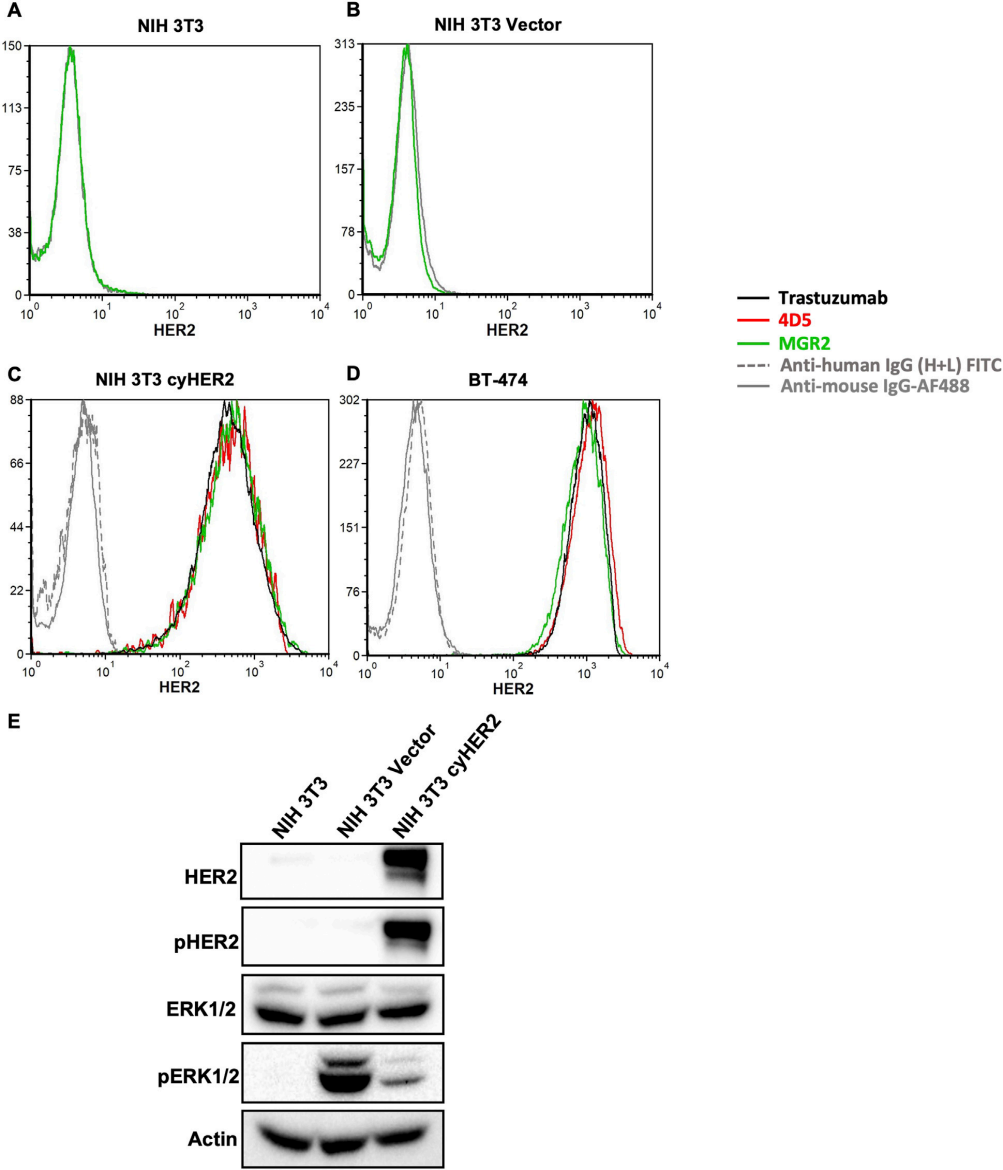
Induction of cyHER2 in NIH 3T3 cells. **(A,B)** Representative profiles of HER2 expression as measured by cytofluorimetric analysis of NIH 3T3 parental cell line and NIH 3T3 Vector cells. Green profile: anti-HER2 monoclonal antibody MGR2; gray profile: anti-mouse IgG AF488. **(C,D)** Cytofluorimetric analysis on NIH 3T3 cyHER2 and BT-474 cell lines of different anti-human HER2 monoclonal antibodies: red profile, 4D5; green profile, MGR2; black profile, trastuzumab; grey profiles, secondary antibodies. **(E)** Western blot bands for HER2 and ERK1/2 total and phosphorylated proteins, which showed significantly increased phosphorylated/total ratios compared to parental control (data not shown). Actin was used as loading control; immunoblots were processed with indicated antibodies. Protein panels representative of n = 2-4 experiments.

Cytofluorometric analysis showed that NIH 3T3 transfected cells (referred to as NIH 3T3 cyHER2) achieved a high expression of HER2, comparable to that of reference BT-474, a human HER2+++ breast cancer cell line (Figures 1C,D). Neither the parental cell line nor the transfection control showed cyHER2 expression (Figures 1A,B).

Monoclonal antibodies (mAbs) specifically targeting cynomolgus HER2 are not available. Thus, to evaluate the induction of cyHER2 expression and its cross reactivity with monoclonal antibodies directed against human HER2 (hHER2), a panel of anti-HER2 mAbs was used. As shown in Figures 1C,D, trastuzumab, 4D5 (the murine monoclonal antibody precursor of trastuzumab) and MGR2 effectively bound cyHER2 on transfected cells, as measured by cytofluorimetric analysis.

HER2 activation and signaling were evaluated by Western blot analysis. NIH 3T3 cyHER2 cells showed increased expression of pHER2 and pERK1/2 as compared to the parental cell line, suggesting the activation of HER2 and its downstream signaling pathways. pERK1/2 increase in the transfection control could be an effect of the transfection process (Figure 1E) (Stepanenko and Heng, 2017).

### Neoplastic transformation of cynomolgus HER2 transfectants

**FIGURE 2:**
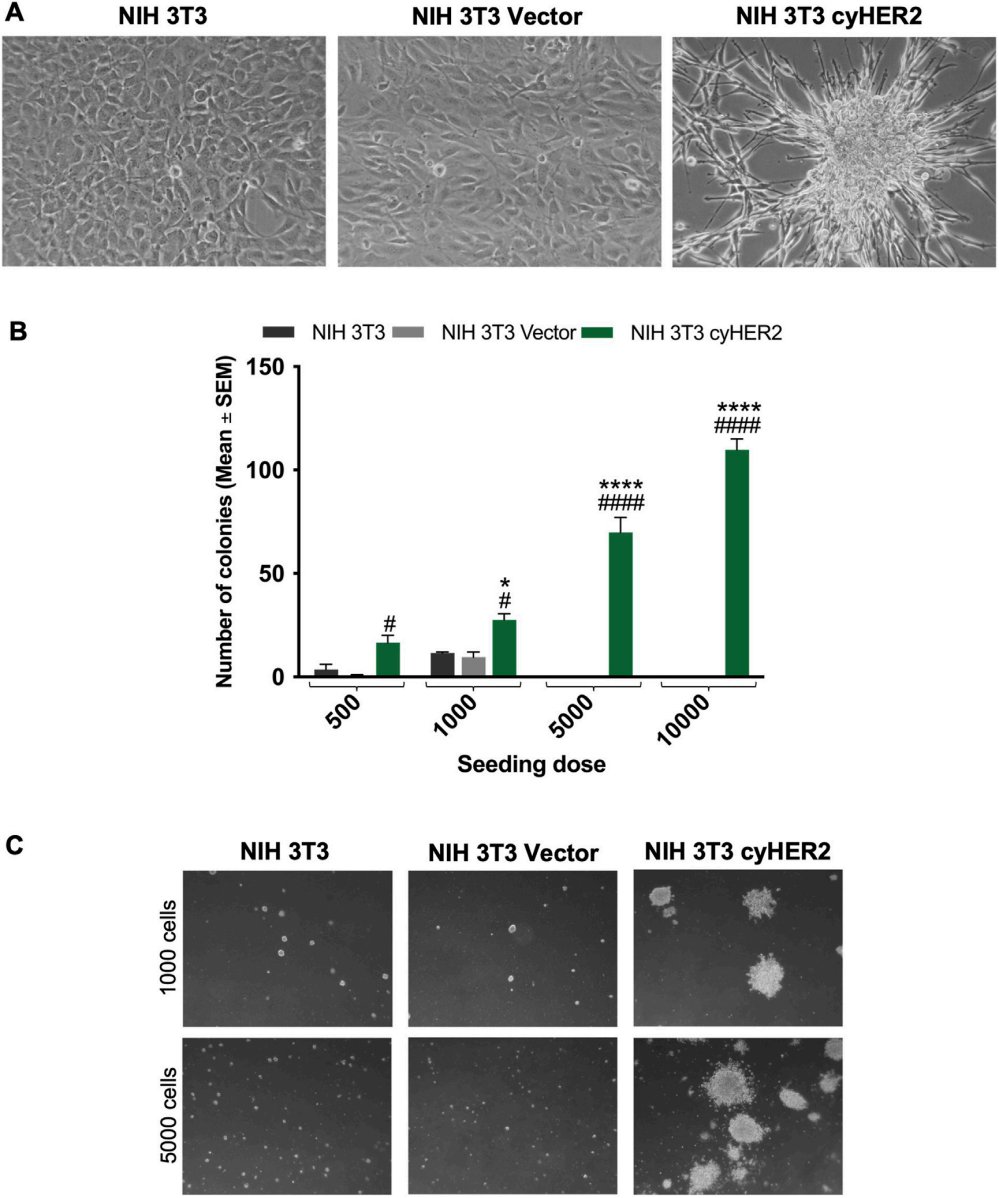
Characterization of cyHER2-expressing cells. **(A)** Representative micrograph of cell lines cultured *in vitro*. **(B)** 3D-soft agar cell growth capability of NIH 3T3 cyHER2 and controls. Each bar represents the mean ± SEM number of colonies larger than 90 μm counted with the aid of a micrometer, 24 days after seeding; *p < 0.05, ****p < 0.0001 at least NIH 3T3 cyHER2 vs. NIH 3T3; #p < 0.05, ####p < 0.0001 at least NIH 3T3 cyHER2 vs. NIH 3T3 Vector, by two-way ANOVA Bonferroni’s multiple comparison test. **(C)** Representative micrographs of live agar colonies of NIH 3T3, NIH 3T3 Vector and NIH 3T3 cyHER2 at indicated seeding doses (dark-field, Lens 2.5X, Eyepiece 12.5X).

CyHER2 expression was associated with morphological changes: confluent transfected cells showed a morphology resembling transformation foci and losing contact inhibition (Di Fiore et al., 1987; Hudziak et al., 1987), whereas the transfection control did not show morphological changes (Figure 2A). Therefore, we analyzed the transformed phenotype of NIH 3T3 cyHER2 cells. CyHER2 expression significantly enhanced soft agar 3D colony formation ability of NIH 3T3 cells. While the parental cell line and the transfection control did not form colonies, NIH 3T3 cyHER2 efficiently grew in 3D-soft agar condition, forming a mean of 70 ± 7 colonies (mean ± SEM, Standard Error of Mean) 24 days after the seeding of 5,000 cells/well (Figures 2B,C).

### Sensitivity to HER2 targeted agents

The development of a cellular model expressing cynomolgus HER2 represent a potent tool to obtain predictive preclinical information on anti-human HER2 therapeutic agents. Therefore, we tested NIH 3T3 cyHER2 sensitivity to already approved human-anti HER2 agents.

### Trastuzumab

**FIGURE 3:**
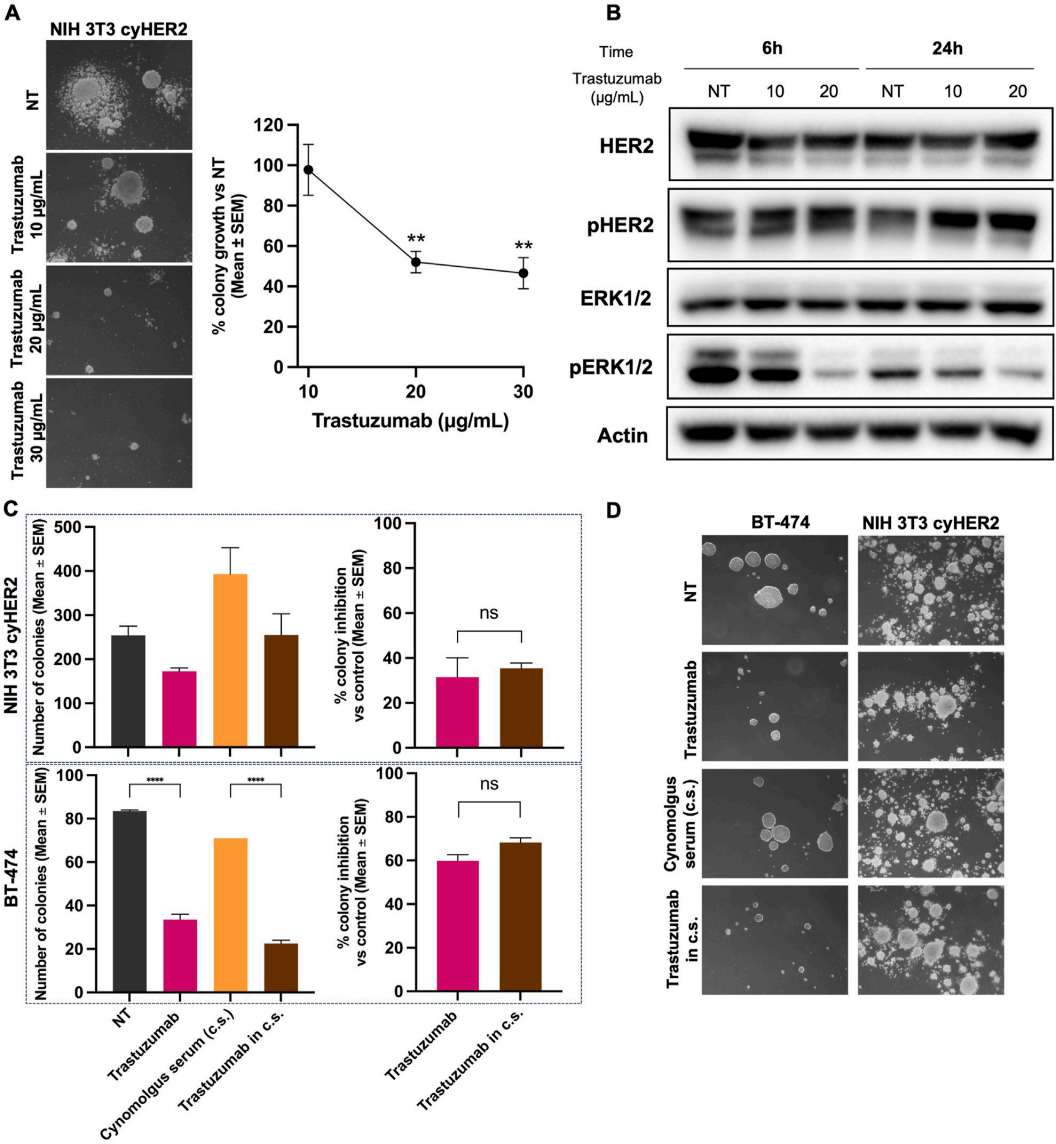
Inhibition of NIH 3T3 cyHER2 3D agar colony growth by trastuzumab. **(A)** Left panel, representative micrographs of live agar colonies were taken with an inverted microscope (dark-field, Lens 2.5X, Eyepiece 12.5X). Right panel, percentage of 3D-colony growth vs. untreated (NT) of NIH 3T3 cyHER2 treated with trastuzumab at indicated concentrations (10, 20 and 30 μg/mL). Each dot represents the mean ± SEM of percentage of colony growth vs. NT (n = 4) **p < 0.01 vs. control by one-way Tukey’s multiple comparison test. Colonies >90 μm were count with an ocular micrometer in dark-field; Untreated (NT) yielded to a mean of 83.75 ± 26.24 colonies (mean ± SEM). **(B)** Western blot analysis for total and phosphorylated HER2 and ERK1/2 in NIH 3T3 cyHER2 cells at 6 and 24 h after trastuzumab treatment (at 10 and 20 μg/mL). Actin was used as housekeeping control and protein expression levels were normalized on relative actin expression. Immunoblots were processed with indicated antibodies, proteins panel is representative of n = 3 experiments. **(C)** Inhibitory effect of trastuzumab in presence and absence of cynomolgus sera (c.s.) on 3D-colony growth of NIH 3T3 cyHER2 and BT-474. Histograms report the mean ± SEM of number of colonies >90 µm and the percentage of colony growth inhibition of both cell lines, left and right panels respectively. ****p < 0.0001 vs. control by one-way Tukey’s multiple comparison test (n = 2). **(D)** Representative micrographs of live NIH 3T3 cyHER2 and BT-474 colonies under indicated treatments (dark-field, 2.5X, Eyepiece 12.5X).

Three-dimensional (3D) cell cultures were found to better reveal the tumor-inhibitory activity of monoclonal antibodies (Kapałczyńska et al., 2016). Thus, to evaluate the functional activity of trastuzumab on cyHER2 expressing cells, a 3D soft-agar colony growth inhibition assay was performed. Trastuzumab at 20 and 30 μg/mL significantly impaired 3D colony formation of NIH 3T3 cyHER2 cells, reducing the number of colonies by 48% and 53% respectively, compared to untreated cells (NT, Figure 3A). NIH 3T3 parental cell line did not show colony growth and, consequently, its sensitivity to trastuzumab treatment could not be evaluated (Supplementary Figure S3).

The effects of trastuzumab treatment on HER2 signaling were evaluated by Western blot, 6 and 24 h after antibody treatment. While trastuzumab did not induce any changes in HER2 activation state, a decrease in pERK1/2 expression was detected at 6 h of trastuzumab 20 μg/mL treatment and at 24 h of trastuzumab 10 and 20 μg/mL (Figure 3B).

To further characterize NIH 3T3 cynomolgus HER2 *in vitro* model, we decided to test NIH 3T3 cyHER2 3D growth in the presence of cynomolgus serum, with the aim to better mimic the microenvironment context. Cynomolgus serum exerted a significant stimulatory growth effect on NIH 3T3 cyHER2 3D-agar colonies growth, increasing the number of colonies by 158% as compared to untreated (NT). Nevertheless, trastuzumab confirmed its functional inhibitory efficacy also in presence of cynomolgus serum, impairing colony growth by 36% compared to cynomolgus serum only (Figures 3C,D).

The human HER2+++ breast cancer cell line BT-474 was used as a positive control of sensitivity to anti-HER2 drugs, as this cell line is extensively employed to study cellular growth and signaling responses to HER2-targeted therapies (Stuhlmi et al., 2015; Brockhoff et al., 2007). As shown in Figures 3C,D, BT-474 colony growth was slightly inhibited by the presence of cynomolgus serum and the number of BT-474 3D-colonies was reduced by 60% by trastuzumab.

### Tucatinib

**FIGURE 4:**
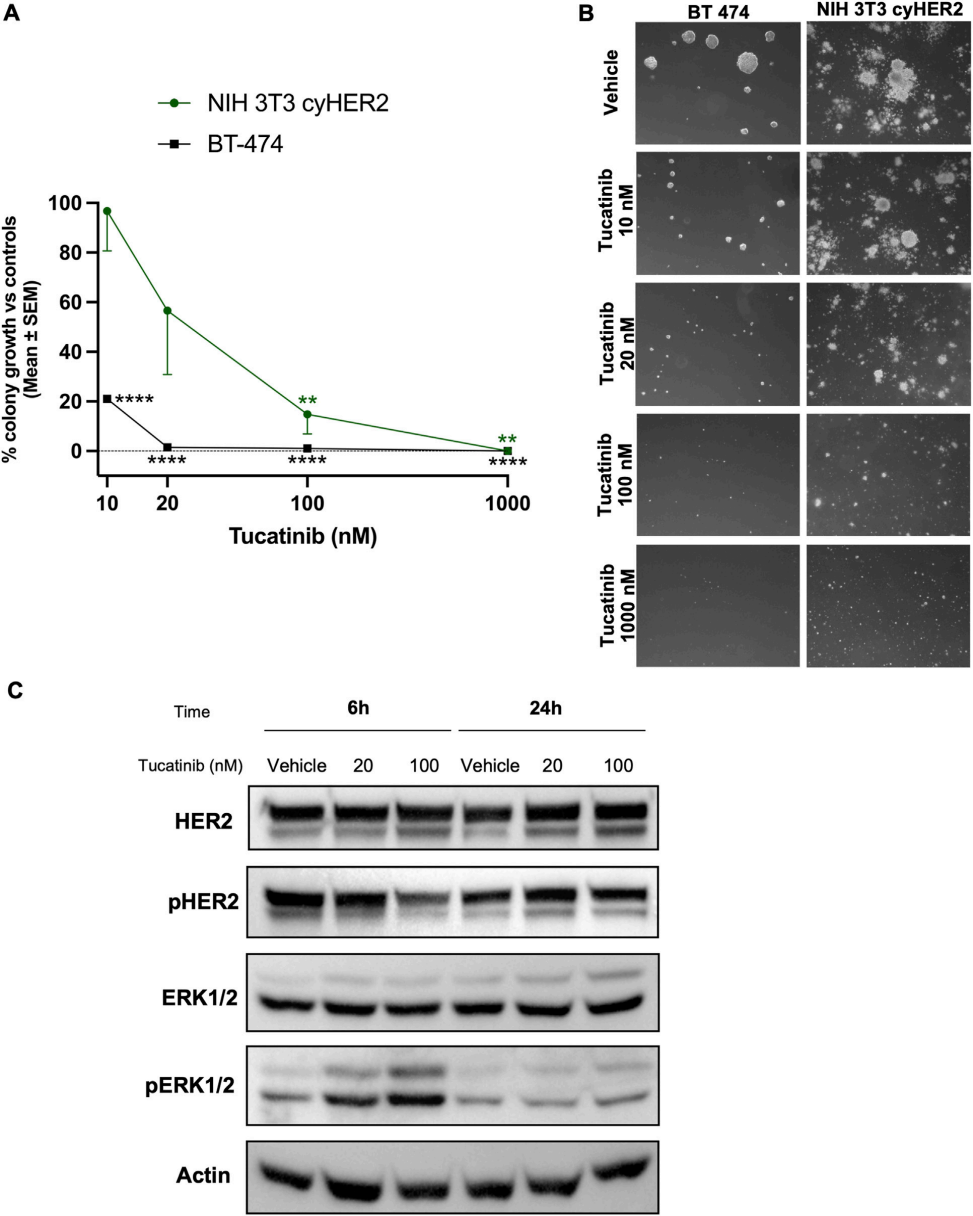
Inhibition of NIH 3T3 cyHER2 3D agar colony growth by tucatinib. **(A)** Percentage of 3D-colony growth vs. controls of NIH 3T3 cyHER2 (green, round dot) and BT-474 (black, square dot) treated with tucatinib at indicated doses. Controls represented the mean between untreated and vehicle (DMSO 0.01%). Each dot represents the mean ± SEM of the percentage of colony growth vs. controls (n = 4 for NIH 3T3 cyHER2 and n = 2 for BT-474). **p < 0.01, ***p < 0.001 and ****p < 0.0001 vs. control, by one-way ANOVA, Dunette’s multiple comparison test. Colonies >90 μm were counted with an ocular micrometer in dark-field; controls corresponded to 50.5 ± 0.5 colonies. **(B)** Representative micrographs of BT-474 and cyHER2-transfected NIH 3T3 live agar colonies under indicated treatments were taken with an inverted microscope (dark-field, Lens 2.5X, Eyepiece 12.5X). **(C)** Western blot analysis for total and phosphorylated HER2 and ERK1/2 in NIH 3T3 cyHER2 cells at 6 and 24 h after tucatinib treatment (at 20 and 100 nM). Actin was used as housekeeping control for protein expression levels normalization. Immunoblots were processed with indicated antibodies, proteins panel is representative of n = 2-4 experiments.

Tucatinib is an oral ATP-competitive tyrosine kinase inhibitor (TKI), with a >50-fold selectivity for HER2 over EGFR (Moulder et al., 2017; Kulukian et al., 2020; O’Brien et al., 2022). NIH 3T3 cyHER2 demonstrated to be sensitive to tucatinib showing a significant 3D colony growth inhibition of 43, 85% and 100% at 20, 100 and 1,000 nM respectively, compared to vehicle (Figures 4A,B). BT-474 colony growth was also significantly inhibited by tucatinib, starting from the 10 nM dose, with a colony growth inhibition of 79% over vehicle (Figures 4A,B).

Western blot analysis revealed a decreased HER2 activation compared to vehicle after tucatinib 100 nM treatment at either 6 and 24 h from treatment in NIH 3T3 cyHER2 cell line. In contrast, no reduction in HER2 activation and signaling was observed at 20 nM. Surprisingly, pERK1/2 appeared to increase with tucatinib treatment at 20 and 100 nM after 6 h from treatment (Figure 4C).

### Induction of cynomolgus HER2 expression in CYNOM-K1 cell line

To establish a cynomolgus HER2-expressing model from cynomolgus cells, CYNOM-K1 cell line was transfected with the same cyHER2 vector previously employed for NIH 3T3 cells. CYNOM-K1 Vector was obtained by the transfection with an empty vector.

**FIGURE 5:**
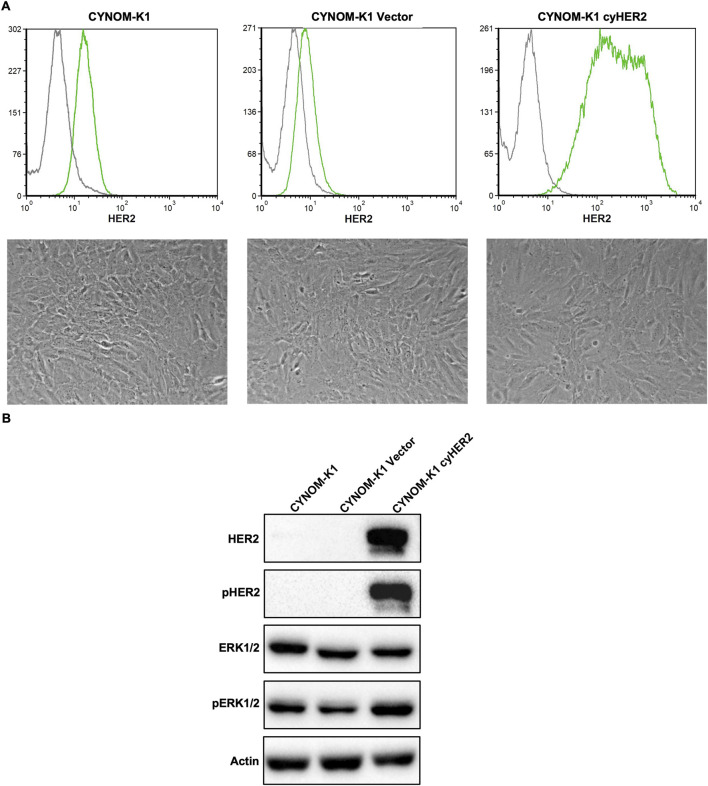
Induction of cyHER2 in CYNOM-K1 cells. **(A)** Upper panels, representative profiles of HER2 expression as measured by cytofluorimetric analysis. Green profile: anti-HER2 monoclonal antibody MGR2; gray profile: anti-mouse IgG AF488. Lower panels, representative micrographs of cell lines cultured *in vitro*. **(B)** Western blot analysis for phosphorylated and total HER2 and ERK1/2 proteins. Actin was used as housekeeping control. Immunoblots were processed with indicated antibodies, proteins panel is representative of n = 3-4 experiments.

CYNOM-K1 cyHER2-transfected cells (referred to as CYNOM-K1 cyHER2) showed a stable HER2 expression, slightly lower than that of NIH 3T3 cyHER2 (see Figures 1, 5), as showed by cytofluorometric analysis. In contrast, both parental and transfection control cell lines showed very low HER2 positivity (Figure 5A upper panels).

Unlike NIH 3T3 cyHER2, CYNOM-K1 cyHER2 showed no morphological changes as compared to parental and transfection control cell lines (Figure 5A lower panels). Nonetheless, Western blot analysis revealed increased pHER2 and pERK1/2 expression compared to controls (Figure 5B).

CyHER2 transfection did not enhance CYNOM-K1 growth capability. CYNOM-K1 cyHER2 were unable to produce colonies in soft agar condition (data not shown), thus precluding the possibility to perform functional 3D tests with anti-HER2 agents on this model.

## Discussion

We developed novel cellular models expressing cynomolgus HER2 (cyHER2) to assess the cross-reactivity of anti-HER2 therapies and their potential impact on non-cancerous cells before proceeding to animal studies.

3D platforms better replicate the tissue-like growth, and the physiological environment compared to bi-dimensional models and can provide a more realistic context for evaluating the inhibitory activity of anti-HER2 agents, overcoming limitations observed in 2D settings (Pickl and Ries, 2009; İpek et al., 2023), therefore we focused our attention on obtaining cyHER2-expressing cells able to grow *in vitro* 3D-soft agar condition.

For this purpose, two cell lines, NIH 3T3 (mouse fibroblasts) (Iwamoto et al., 1985; Leibiger et al., 2013) and CYNOM-K1 (cynomolgus fibroblasts) (Dotti et al., 2017) were transfected with lentiviral particles carrying the cynomolgus *ErbB2* gene to induce stable expression.

Unfortunately, despite stable HER2 expression and signalling activation, CYNOM-K1 cyHER2 cells did not show HER2 addiction and neoplastic transformation features (e.g., 3D growth ability acquisition), precluding the possibility of further using this cell line for our purpose. Nevertheless, further attempts with alternative cynomolgus cell lines might provide better results.

Unlike CYNOM-K1, NIH 3T3 transfected cells exhibited a stable cyHER2 expression and, as previously reported for human HER2 expression (Di Fiore et al., 1987; Hudziak et al., 1987), a transformed phenotype, producing transformation foci in 2D and acquiring 3D growth capability.

Trastuzumab, a monoclonal antibody commonly used in clinical practice (Hudis, 2007; Slamon et al., 2011), and tucatinib, a highly selective anti-HER2 tyrosine kinase inhibitor (TKI) (Olson et al., 2023), were selected as representative anti-HER2 drugs to validate the sensitivity of our model. In 3D-soft agar colony growth assay, trastuzumab significantly impaired the colony formation of NIH 3T3 cyHER2 cells at 20 μg/mL and 30 μg/mL. To create an experimental setup that could as much as possible resemble the physiological conditions in which cynomolgus cells are found, cynomolgus monkey serum was added to the agar matrix. Unexpectedly, monkey serum significantly increased colony growth of NIH 3T3 cyHER2 cells without altering their sensitivity to trastuzumab. Trastuzumab inhibition was accompanied by a down-regulation of ERK1/2 activation, as reported for human HER2-positive cell line models (Bagnato et al., 2017). No effects on HER2 and pHER2 were observed at the analysed timepoints, in line with what was observed in human cell lines (Kostyal et al., 2012; Maadi et al., 2018).

Tucatinib is a specific and reversible inhibitor of the protein tyrosine kinase activity of HER2. Unlike other TKIs, such as lapatinib and neratinib, it exerts minimal inhibition on EGFR and it is showing promising therapeutic and safety results both as a single agent and in combination with trastuzumab (Moulder et al., 2017; Kulukian et al., 2020; Sirhan et al., 2022; Criscitiello et al., 2023). We evaluated the sensitivity of our model to tucatinib selecting a dose range comparable to those observed in patients’ sera following tucatinib treatment (Olson et al., 2023; Topletz-Erickson et al., 2022). NIH 3T3 cyHER2 colony formation in 3D-soft agar condition was inhibited by 40% and 80% by tucatinib 20 and 100 nM, respectively. An increase in ERK1/2 was also observed after treatment. This pronounced activation of ERK1/2 could be associated with both pro-survival or pro-apoptosis cell responses induced by the treatment (Smirnova et al., 2010; Li et al., 2014; Sugiura et al., 2021).

Both in the case of trastuzumab and tucatinib, we observed that the sensitivity of our model was reduced if compared to the response of the human HER2+++ breast cancer cell line BT-474 (halved for trastuzumab and three times lower for tucatinib).

Additionally, the median fluorescence intensity of three different anti-HER2 antibodies (trastuzumab, 4D5, and MGR2) binding to cynomolgus HER2 was lower compared to the one observed for human HER2 in BT-474 cells. Notably, a further improvement will aim to calculate binding affinities obtained from surface plasmon resonance (SPR) or biolayer interferometry (BLI) to provide a more detailed characterization of monoclonal antibodies–cyHER2 interactions.

The alignment of human and cynomolgus *ErbB2* sequences revealed an identity percentage of 98.25% and identified several non-conserved amino acids between the two species, with 6 out of 22 amino acids located in the extracellular domain (ECD) of HER2 protein and 12 out of 22 in the intracellular domain (ICD) (Supplementary Figure S1). However, despite the high degree of homology and the extent of identity percentage between human and cynomolgus HER2 sequence, the small differences in the amino acid sequence, in particular in the ECD, might affect monoclonal antibody and TKI binding. The human HER ECD (human residues 23–652) shows a high degree of sequence identity to the cynomolgus one, and the four structural HER2 subdomains (I–IV) are highly conserved in length and amino acids pattern (as represented in Supplementary Figure S1). Importantly, the residues that comprise the trastuzumab epitope are represented by the juxtamembrane loop segments in subdomain IV. In particular, the antigen-antibody interactions are mediated by three loop regions along the domain IV C-terminal portion: the first region is represented by a loop made of amino acid residues 557-561, the second is formed by residues 570-573 and the third from residues 593-603 (Cho et al., 2003; Khoury et al., 2011; Deng et al., 2014). Although the identified non-conserved residues are not directly comprised in these loop regions, small changes induced by these non-conserved amino acids in the ECD may anyway affect the strength of the antibody-antigen interaction. Indeed, the binding interactions made between the antibody and the receptor are mainly electrostatic for the first and the third loops, instead of the second loop which made mainly hydrophobic interactions (Cho et al., 2003; Khoury et al., 2011). Studies demonstrated that the ability of trastuzumab to bind to these regions directly correlates to its efficacy in achieving therapeutic effects and, interestingly, the antibody binding to an antigen are mostly dependent upon non-covalent interactions (i.e., hydrogen bonds, electrostatic forces and van der Waals interactions) (Khoury et al., 2011). The key residues mediating TKI activity are shown to be located in the hinge region (Ala751–Met801), αC-helix (Lys753–Glu770), catalytic loop (Asp863–Phe864), gatekeeper position (Thr798) and activation loop (Asp885) in the intracellular domain (ICM). These residues are involved in the hydrogen-bonding and hydrophobic interactions, anchoring the small molecule in the ATP-binding cleft. According to our data, these residues are fully conserved in human and cynomolgus HER sequences. These 12 non-conserved amino acids in cynomolgus HER2 ICD are located in the distal C-lobe (beyond approximately residue 900) or in the C-terminal regulatory tail and they do not directly contact tucatinib, unlikely affecting its binding affinity (Wood et al., 2004; Gaibar et al., 2020; Zheng et al., 2024; Sonar et al., 2022). However, they may influence kinase dynamics, causing differences in kinase conformational flexibility, or regulatory phosphorylation, potentially modifying downstream signaling or allosteric stability. Generally, also small changes in the amino acid sequence in the ECD may alter the three-dimensional structure of the epitope recognized by antibodies or other agents, alter dimers formation and impair the binding of the molecule to the target, thus reducing or abolishing its functional activity (Ciftci et al., 2023). We therefore conclude that the structural bases for trastuzumab and tucatinib binding are preserved between these species, consistent with the cross-reactivity observed in our functional assays; although, the presence of non-conserved amino acids in the cynomolgus sequence may cause minor structural alterations which influence the strength of trastuzumab-HER2 binding or differences in the tucatinib-HER2 complex activity, thus hampering their therapeutic effects. This aspect could explain the lower binding of the anti-HER2 monoclonal antibodies to the cynomolgus HER2 expressing cell line and the subsequent reduced sensitivity to anti-HER2 drugs if compared to the HER2+++ human cell line used as positive control. Further investigations, also involving additional types of anti-HER2 drugs, are necessary to clarify this phenomenon and to better characterize our model.

The relevance of animal models in anti-HER2 cross-reactivity studies remains a subject of debate (Atkins et al., 2020). Some studies reported that trastuzumab-induced cardiotoxicity studies in rodents may not be informative since trastuzumab specifically binds to human HER2 and does not cross-react with HER2 in other species (Crone et al., 2002; L et al., 2022). However, Dokter et al. showed that trastuzumab binds to cynomolgus HER2 with an affinity comparable to that of human HER2, making cynomolgus monkey the most relevant species for preclinical studies (Dokter et al., 2014).

The use of nonhuman primate models is even more significant for studies involving anti-HER2 vaccines, which induce strong polyclonal antibody responses (Palladini et al., 2018; Ruzzi et al., 2022; Scalambra et al., 2025). Polyclonal antibodies, which recognise multiple epitopes of a single antigen, are more prone to overcome small structural variations in the target antigen (Lipman et al., 2005; Ascoli and Aggeler, 2018). Three polyclonal antibodies targeting different HER2 extracellular domains (full-length, domain I + II, or domain III + IV) were found to be highly reactive to cyHER2, comparable to human HER2 (Hosseini et al., 2017). Consequently, cynomolgus monkeys represent a highly appropriate model for evaluating polyclonal immune responses, such as those induced by vaccine, against HER2, as well as for studying monoclonal antibody-based therapies and their associated toxicities.

While the cynomolgus HER-2 expressing cellular model described in this work represents a promising tool for initial *in vitro* preclinical toxicity testing of anti-HER2 molecules and polyclonal antibodies, it is important to note that it currently cannot reliably predict the cardiotoxicity typically associated with these therapies. This limitation arises because cardiotoxic effects typically involve complex interactions with cardiac tissue, in particular with cardiomyocytes and their microenvironment, which are not taken into account in this model. Therefore, additional systems incorporating cardiac cells and microenvironment conditions would be necessarily added to asses cardiotoxic risk (e.g., the employment of stem cell-derived cardiomyocytes (Kurokawa et al., 2018)). Nonetheless, the NIH 3T3 cyHER2 model remains a valuable tool for early-stage toxicity screening before proceeding to *in vivo* studies. The establishment of stable cynomolgus HER2-expressing cellular models also provides an essential instrument for translational studies, highly relevant in the development of novel anti-HER2 agents or approaches. In recent years, HER2 has become a key molecular target not only for conventional targeted therapies (i.e., monoclonal antibodies, tyrosine kinase inhibitors or antibody-drug conjugates) but also for emerging advanced technologies, like nanotechnology-based drug delivery platforms. For example, recent studies have demonstrated the potential of integrating HER2-targeted monoclonal antibodies into polymeric nanoscale drug-delivery systems (Sakhi et al., 2022; Liu et al., 2023; Caputo et al., 2023; Selepe et al., 2024; Vanni et al., 2025), which could exploit 3D *in vitro* models (like the NIH 3T3 cyHER2 model) for species cross-reactivity studies, which otherwise can be evaluated only through non-functional techniques like the ELISA assay. Consequently, the development of stable and reliable cynomolgus HER2-expressing cellular model could provide a valuable translational bridge for preclinical assessment. Such model could provide a worthwhile experimental platform not only in the evaluation of pharmacological cross-reactivity of therapeutic agents, but also could support and facilitate the optimization of modern delivery systems, accelerating clinical translation.

## Conclusion

Animal experimentation remains a necessary step in biomedical research despite ongoing advancements in alternative methods. Nonhuman primates are indispensable animal models due to their high genetic homology and physiological similarity to humans. However, their use is strictly regulated and limited, and alternative models are needed to reduce animal involvement in preclinical studies. In this context, the 3D cynomolgus monkey (*Macaca fascicularis*) HER2 (cyHER2)-expressing cellular model presented in this work represents a promising tool for initial preclinical toxicity tests or screening of anti-HER2 therapies before proceeding to *in vivo* studies, aligning with the 3Rs principle.

## Data Availability

The datasets presented in this study can be found in online repositories. The names of the repository/repositories and accession number(s) can be found below: https://www.ncbi.nlm.nih.gov/, https://www.ncbi.nlm.nih.gov/gene/102146608/, https://www.ncbi.nlm.nih.gov/gene/2064.

## Glossary

2D: two-dimensional
3D: three-dimensional
3Rs: replacement, reduction and refinement
ADC: antibody–drug conjugate
BLAST: Basic Local Alignment Search Tool
CHF: congestive heart failure
CMV: cytomegalovirus
cyHER2: cynomolgus HER2
DMEM: Dulbecco’s Modified Eagle Medium
ECD: extracellular domain
EDTA: Ethylenediaminetetraacetic acid
EGFR: epidermal growth factor receptor
ELISA: enzyme-linked immunosorbent assays
EMEM: Eagle’s Minimum Essential Medium
ERBB2: erb-b2 receptor tyrosine kinase 2
FBS: fetal bovine serum
HER2: human epidermal growth factor receptor 2
hHER2: human HER2
ICD: intracellular domain
LVEF: left ventricular ejection fraction
LVSD: left ventricular systolic dysfunction
mAbs: monoclonal antibodies
MOI: multiplicity of infection
NEAA: Non-Essential Amino Acids
NHP: nonhuman primates
NT: untreated
RPMI: Roswell Park Memorial Institute Medium
SEM: Standard Error of Mean
TKI: tyrosine kinase inhibitor
TMD: transmembrane domain
