# Supplementary material for: Development of cellular models expressing cynomolgus (Macaca fascicularis) HER2 for the functional evaluation of cross-reactive anti-human HER2 response
Source: Front Pharmacol. 2025 Nov 13;16:1675875. doi: 10.3389/fphar.2025.1675875 (PMC12657493; doi:10.3389/fphar.2025.1675875)
Supplement: Supplementary file 1 [file DataSheet1.pdf]

Supplementary Figures

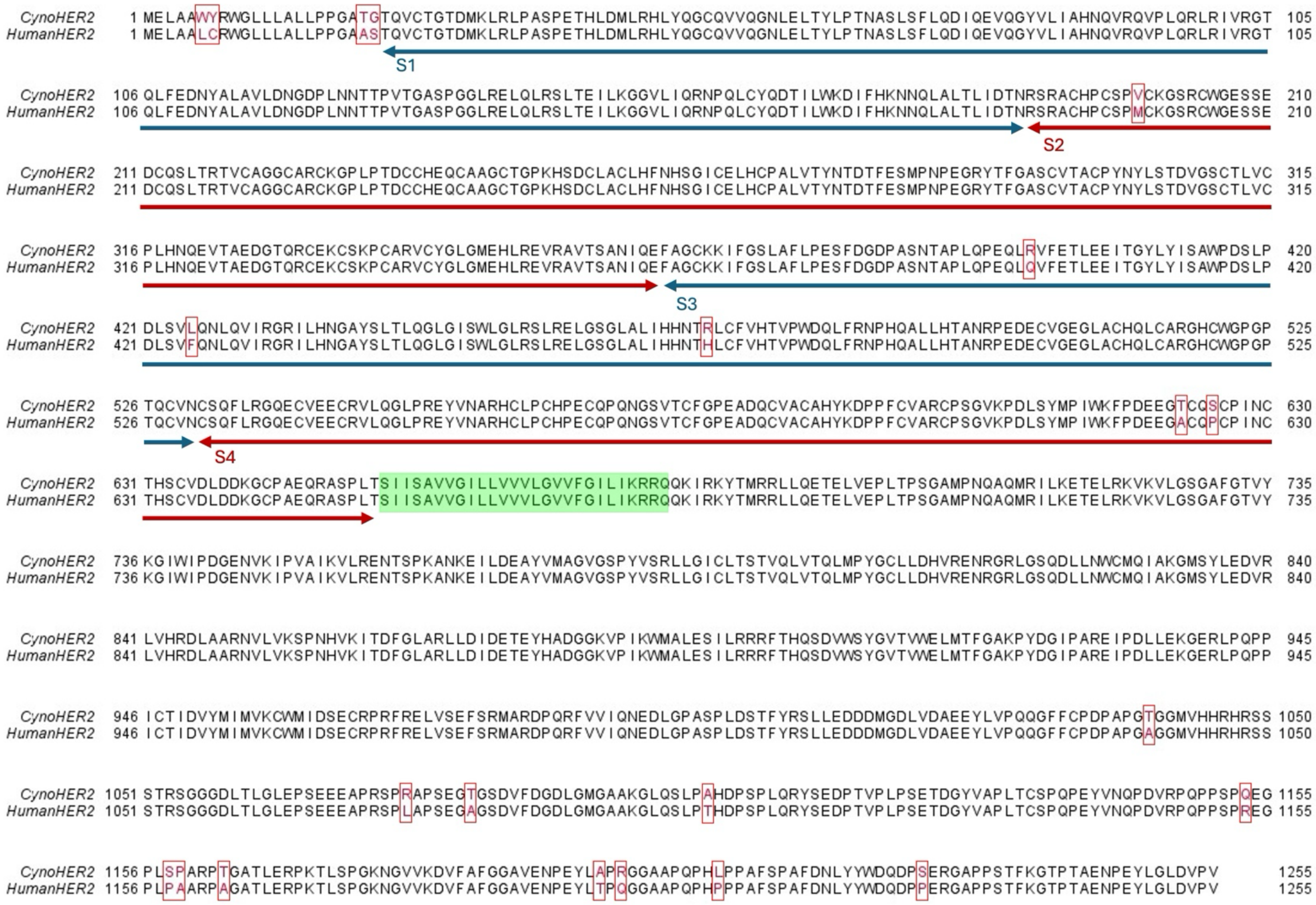

Supplementary Figure 1. Amino acid pairwise alignment for cynomolgus monkey (CynoHER2/1-1255) and human (HumanHER2/1-1255) HER2 obtained with Jalview. Non-colored regions represent conserved amino acids and red colored and squared amino acids represent non-conserved amino acids between human and cynomolgus HER2 sequences. Green highlighted amino acids represent the transmembrane domain (TMD, from amino acids 653 to 675), dividing the extracellular domain (ECD, from amino acids 23 to 652) and the intracellular domain (ICD, from amino acids 675 to 1255) reported from UniProt. The extracellular domain is further subdivided into four domains, consisting in two L domains (domains I and III, respectively S1 and S3 indicated in blue) and two cysteine-rich domains (domains II and IV, respectively S2 and S4 in red). The UniProt Consortium Database P04626 ErbB2 human, https://www.uniprot.org/ accessed March 13, 2025.

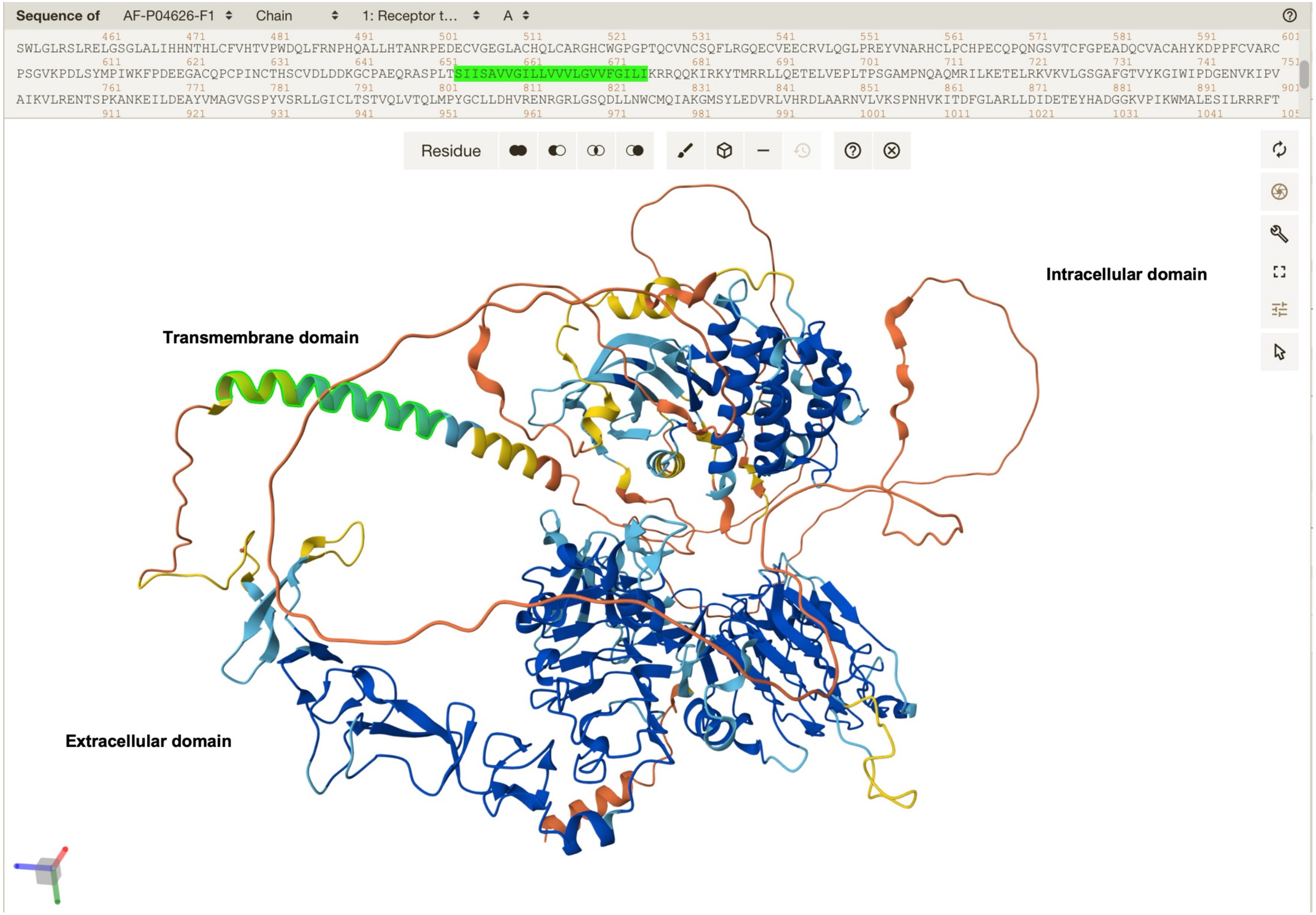
Sequence of AF-P04626-F1 Chain 1: Receptor t... A
461 471 481 491 501 511 521 531 541 551 561 571 581 591 601
SWLGLRSLRELGSGLALIHHNTHLCFVHTVPWDQLFRNPHQALLHTANRPEDECVGEGLACHQLCARGHCWGPGPTQCVNCSQFLRGQECVEECRVLQGLPREYVNARHCLPCHPECQPQNGSVTCFGPEADQCVACAHYKDPPFCVARC
611 621 631 641 651 661 671 681 691 701 711 721 731 741 751
PSGVKPDLSYMPIWKFPDEEGACQPCPINCTHSCVDLDDKGCPAEQRASPLTSIISAVVGILLVVVLGVVFGILIKRRQQKIRKYTMRRLLQETELVEPLTPSGAMPNQAQMRILKETELRKVKVLGSGAFGTVYKGIWIPDGENVKIPV
761 771 781 791 801 811 821 831 841 851 861 871 881 891 901
AIKVLRENTSPKANKEILDEAYVMAGVGSPYVSRLLGICLTSTVQLVTQLMPYGCLLDHVRENRGRLGSQDLLNWCMQIAKGMSYLEDVRLVHRDLAARNVLVKSPNHVKITDFGLARLLDIDETEYHADGGKVPIKWMALESILRRRFT
911 921 931 941 951 961 971 981 991 1001 1011 1021 1031 1041
Residue
Transmembrane domain
Intracellular domain
Extracellular domain

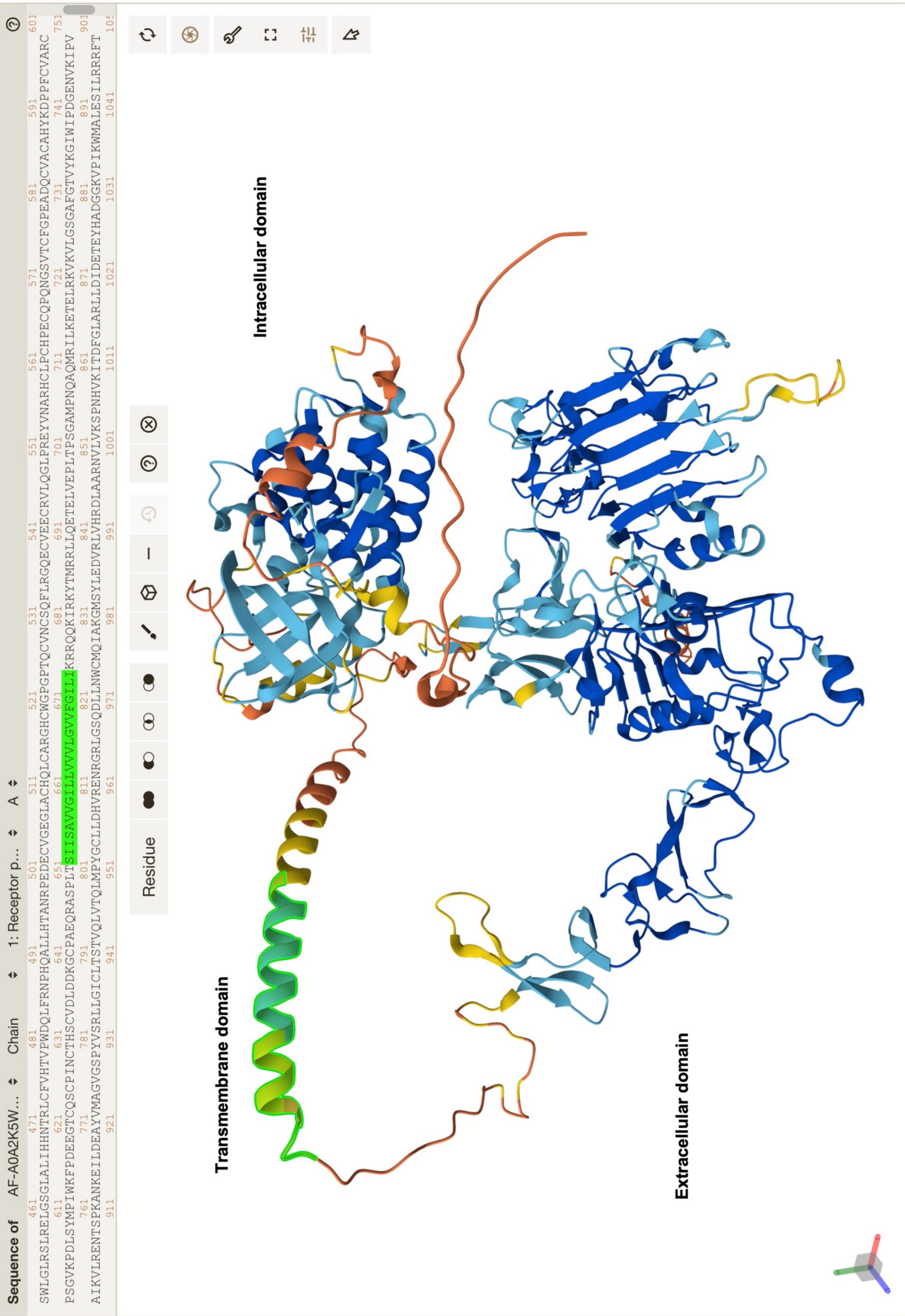
Sequence of
AF-A0A2K5W...
Chain
1: Receptor p...
A
Residue
Intracellular domain
Transmembrane domain
Extracellular domain

Supplementary Figure 2. Human (first panel) and cynomolgus (second panel) HER2 proteins predicted by AlphaFold (https://alphafold.ebi.ac.uk/entry/P04626 and https://alphafold.ebi.ac.uk/entry/A0A2K5WUE3, respectively). The transmembrane domain is highlighted in green to discriminate between the extracellular and intracellular domains in the predicted protein conformations.

A

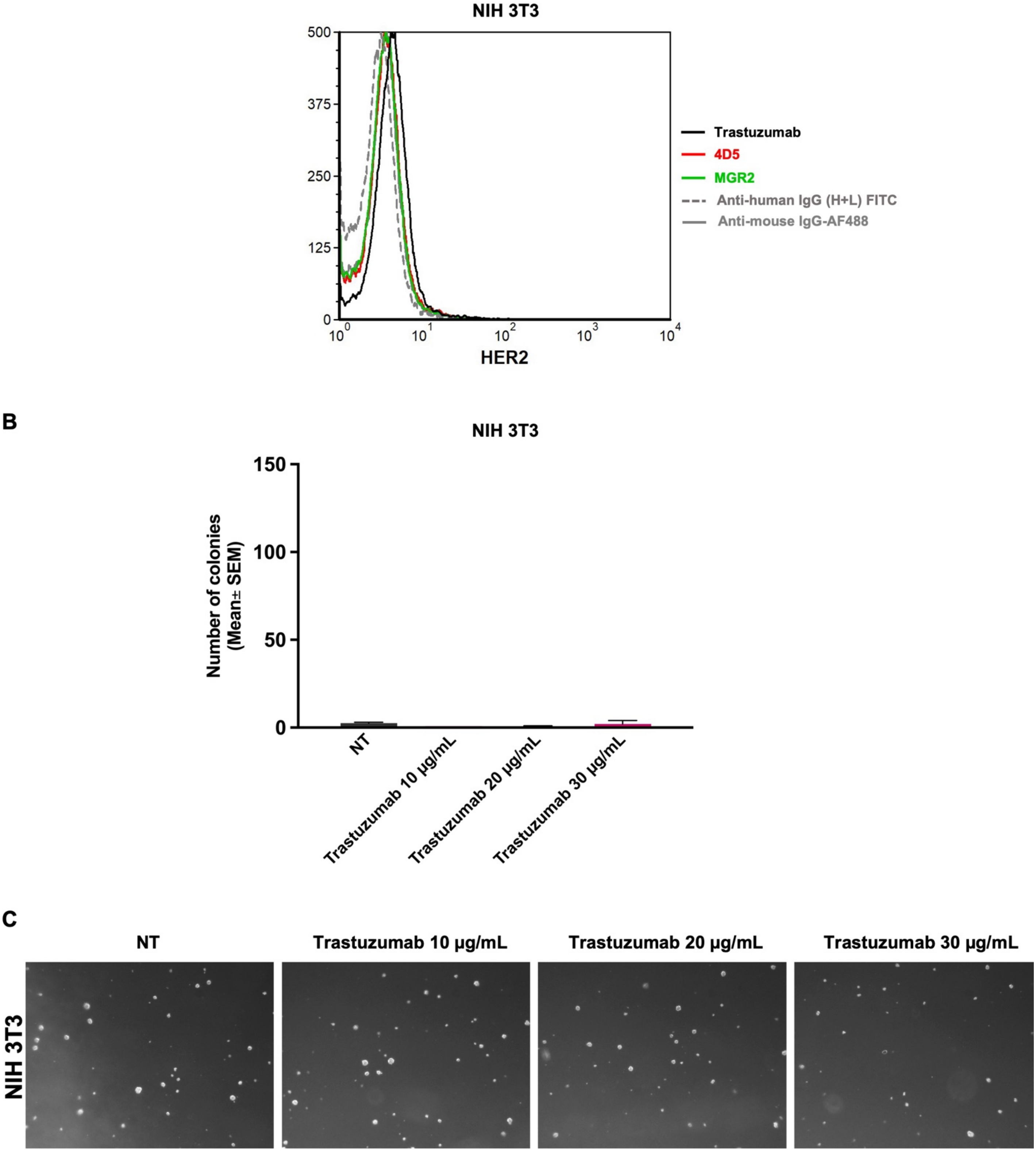

Supplementary Figure 3. A Cytofluorimetric analysis on NIH 3T3 parental cell line of different anti-human HER2 monoclonal antibodies: red profile, 4D5; green profile, MGR2; black profile,

trastuzumab; grey profiles, secondary antibodies. **B** Trastuzumab effect at indicated concentrations on 3D-colony growth of NIH 3T3 parental cell line. For comparison with NIH 3T3 cyHER2 transfected cells, see Figure 3A. Histograms report the mean ± SEM of number of colonies >90µm with an ocular micrometer in dark-field. **C** Representative micrographs of live agar colonies of NIH 3T3 (dark-field, Lens 2.5X, Eyepiece 12.5X).
